# Nothing about us without us: A co‐production strategy for communities, researchers and stakeholders to identify ways of improving health and reducing inequalities

**DOI:** 10.1111/hex.13709

**Published:** 2023-01-22

**Authors:** Alexandra Albert, Shahid Islam, Muki Haklay, Rosemary R. C. McEachan

**Affiliations:** ^1^ Extreme Citizen Science Research Group, Geography Department University College London London UK; ^2^ Bradford Institute for Health Research Bradford Teaching Hospitals NHS Foundation Trust Bradford UK

**Keywords:** Appreciative Inquiry, co‐production, ethnicity, health inequalities, public health, strategy development

## Abstract

**Introduction:**

Co‐production with communities is increasingly seen as best practice that can improve the quality, relevance and effectiveness of research and service delivery. Despite this promising position, there remains uncertainty around definitions of co‐production and how to operationalize it. The current paper describes the development of a co‐production strategy to guide the work of the ActEarly multistakeholder preventative research programme to improve children's health in Bradford and Tower Hamlets, UK.

**Methods:**

The strategy used Appreciative Inquiry (AI), an approach following a five‐step iterative process: to *define* (Step 1) scope and guide progress; to *discover* (Step 2) key issues through seven focus groups (*N* = 36) and eight in‐depth interviews with key stakeholders representing community groups, and the voluntary and statutory sectors; to *dream* (Step 3) best practice through two workshops with AI participants to review findings; to *design* (Step 4) a co‐production strategy building on AI findings and to *deliver* (Step 5) the practical guidance in the strategy.

**Results:**

Nine principles for how to do co‐production well were identified: power should be shared; embrace a wide range of perspectives and skills; respect and value the lived experience; benefits should be for all involved parties; go to communities and do not expect them to come to you; work flexibly; avoid jargon and ensure availability of the right information; relationships should be built for the long‐term; co‐production activities should be adequately resourced. These principles were based on three underlying values of equality, reciprocity and agency.

**Conclusion:**

The empirical insights of the paper highlight the crucial importance of adequate resources and infrastructure to deliver effective co‐production. This documentation of one approach to operationalizing co‐production serves to avert any misappropriations of the term ‘co‐production’ by listening to service users, stakeholders and other relevant groups, to develop trust and long‐term relationships, and build on the learning that already exists amongst such groups.

**Patient or Public Contribution:**

The work was overseen by a steering group (*N* = 17) of individuals, both professional and members of the public with experience in undertaking co‐production, and/or with some knowledge of the context of the two ActEarly field sites, who provided regular oversight and feedback on the AI process.

## 1 INTRODUCTION

This paper reports on the process of co‐producing a co‐production strategy for a large UK consortium of researchers, stakeholders and communities called ‘ActEarly’, which aims to improve the health opportunities of families living in deprived areas. The importance of working in partnership with communities to address issues which impact their health and happiness is well recognized.^1^ Reviews have found that health interventions which engaged communities in their development or delivery have a positive impact on health outcomes.^2^ However, effective partnership working with communities can be challenging, and there can be adverse impacts if opinions are sought, but little change is demonstrated. Co‐production is a ‘complex social phenomenon’, and the relationships between processes and outcomes can be ambiguous; outcomes may include ‘soft’ variables that are ‘hard’ to measure in practice, such as improved trust, shared responsibility, levels of influence and ownership over projects.^3^ Evidence about ‘what works’ or ‘how’ to do co‐production is limited. Smith et al's.^4^ scoping review of co‑production practice and future research priorities in UK‐funded applied health research urges researchers to be clearer in reporting ways in which they are operationalizing co‐production by providing a set of values and operating principles through which co‐production could be implemented.

### 1.1 Context of ActEarly

The ActEarly UK Preventative Research Programme aims to promote health and well‐being in early life in two multicultural areas of the United Kingdom with high rates of child poverty, Bradford in West Yorkshire, and the Borough of Tower Hamlets in East London.^5^ Living in an area with high levels of child poverty often coincides with exposure to other economic, physical, cultural, learning, social and service environmental risk factors, which can predispose children and their families to poorer mental and physical health outcomes. Co‐production is at the heart of ActEarly, launched in 2019, which uses a ‘City Collaboratory’ model to unite communities, researchers and stakeholders, including local governments, the NHS and the third sector to identify, co‐produce and implement system‐wide early life upstream prevention interventions.^5^

### 1.2 The tricky problem of defining co‐production

What unites nearly all researchers and practitioners involved in co‐production is a recognition of the difficulty in defining it. It is noted as a slippery,^6^ woolly^7^ and muddled^8^ concept, the benefits of which may be diminished if the definition is unclear or misapplied. Co‐production and co‐design are conceptualized in a wide range of ways, and the elasticity of the term has been referred to as both its strength and its limitation.^3^ Co‐research has been used as an umbrella term to encompass a family of approaches, such as ‘participatory’, ‘emancipatory’ and ‘inclusive’ research, which reflect a turn towards involving communities in the process of knowledge production.^9^ Co‐research aims to put principles of empowerment into practice, by offering participants greater control over the research process while providing opportunities to learn and reflect upon their experience.^10^ Co‐production builds on this, focusing on the delivery of more responsive, personalized public services in an equal and reciprocal relationship between professionals, people using services, their families and their neighbours.^11^, ^12^, ^13^, ^14^, ^15^ Where activities are co‐produced in this way, there is great potential for both services and neighbourhoods to become far more effective agents of change. These principles are increasingly applied to the production of knowledge, and co‐production is now a mainstream term in health research.^16^ Similarly, the UK National Institute for Health and Care Research (NIHR) see co‐production as an approach in which ‘researchers, practitioners and members of the public work together, sharing power and responsibility from the start to the end of the project, including the generation of knowledge’.^17^ ^,p.1^

Key considerations for effective co‐production include the recognition that it is context‐dependent and that it requires building trust and creating opportunities for genuine power sharing and respect amongst all partners. The approach to co‐production used as a starting point by the ActEarly consortium builds on others' definitions and sees it as a collaborative process involving researchers, practitioners, decision‐makers and the public working together, sharing power and responsibility.^5^

Rather than seeking universal definitions of terms such as co‐production, Masterson et al.^18^ recommend that future applied research should focus on articulating the underlying principles and values that need to be translated and explored in practice. For this reason, this paper sets out the principles and values underpinning co‐production to inform ActEarly's work undertaken with its communities.

### 1.3 Aims and objectives: Co‐producing a co‐production strategy

The aim of this study was to develop a co‐production strategy to inform the work undertaken with these communities within and beyond the ActEarly programme. Working with communities and stakeholders in Bradford and Tower Hamlets, our objectives were to use an asset‐based Appreciative Inquiry (AI) approach to co‐produce a set of guiding principles and core values which would form the basis of the co‐production strategy.

An assets‐based approach was used, which focussed on the positives of what has worked in these areas and by concentrating on existing assets. This was achieved by going to local organizations and community groups in both ActEarly locations and allowing them to guide what should be included in the strategy; the aim being to produce an end product—the strategy report itself—that carries the values embedded within it.

## 2 METHODS

**Figure 1:**
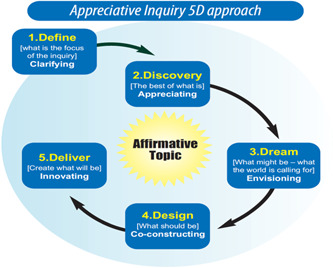
5Ds model of Appreciative Inquiry

The ActEarly Co‐production strategy was designed through the application of AI. A fundamental principle of AI is its focus on assets and strengths within communities rather than focusing on deficits and problems.^19^ The basic assumption is that in every human system, something works right and contains certain elements that make it vital, effective and successful.^20^ AI helps people to focus on what is working well, the positive core and identifying strengths by engaging them in inquiries and stories that highlight and then leverage those strengths.^19^ The deliberately affirmative assumptions of AI about people, organizations and relationships are in stark contrast to more traditional forms of research that seek to analyse or diagnose problems. The AI took the 5D approach (so‐called because it is based on five elements that start with D), which is depicted in Figure 1 below and with each domain described below.

### 2.1 Step 1: *Define*

The research process was overseen by a steering group, made up of 17 individuals (professionals and members of the public) with experience in undertaking co‐production, and/or with some knowledge of the context of the two ActEarly field sites. The steering group provided oversight and feedback on the AI process, and, where necessary, suggested adjustments to the methods proposed. They helped to identify participants adequately reflecting the diversity of the populations across both ActEarly sites.

### 2.2 Step 2: *Discover*

**Figure 2:**
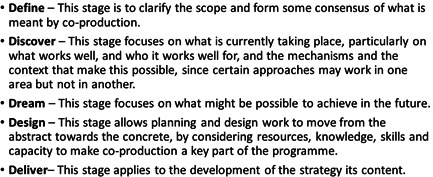
5Ds model explanation text

Seven focus groups (*N* = 36) and eight in‐depth interviews with key stakeholders were undertaken from March to November 2021 across the two ActEarly field sites. The majority of data collection was online, with the exception of two focus groups, and one interview, which was held in person. Participants included stakeholders representing community groups, residents' associations and people employed in community engagement work across the voluntary sector and the statutory sector. A long list of potential participants in each location was identified, most of whom were already known to the research team and the wider work of ActEarly, such as Public and Patient Involvement groups associated with the Bradford Institute for Health Research. We also included groups and networks that may have had limited opportunity to connect with ActEarly. The groups, and individual stakeholders, were approached by the research team via telephone or email and a request was made to organize a session at an appropriate time for the research team to deliver an online workshop or interview. A snowball sampling approach was used, and at the end of each interview or focus group, participants were asked if there were other key stakeholders that should be included in the research. This approach was continued until a saturation point was achieved with the comments and themes being raised in the interviews and focus groups. The topic guides for these discussions were designed around the 5Ds model in Figures 1 and 2. The transcripts and field notes from the focus groups were systematically reviewed by two members of the research team and grouped according to the emergent themes, ideas and concepts. Thematic analysis (TA) was used to identify the preliminary results from this stage of the AI process.

### 2.3 Step 3: *Dream*

The preliminary results of the AI process were then presented at two workshops in November with the patient and public contributors and researchers. Thirty‐five people attended the 2 hour in‐person workshop in Bradford, and 17 people attended the 2 hour online workshop in London. These workshops provided an opportunity to present back to participants the preliminary findings from the AI process and to dream about best practices. The team opened up a discussion with participants about the importance of co‐production to ActEarly, who was engaged during the AI process and preliminary findings. Participants were then asked to reflect on what the team should avoid, what they should eliminate and what they should enhance, or do more of. Following breakout group discussions on these topics, participants were then asked to collaboratively develop one idea that the team could take forward. Discussions at these two workshops informed the next steps of strategy development. Field notes were taken by facilitators attending the workshops, which were supplemented by content recorded in Google Jamboards for the online workshop.

### 2.4 Step 4: *Design and analyse*

The co‐production strategy was designed using data collected from focus groups, interviews and workshops. Conversations were recorded and the transcripts and field notes were systematically reviewed by the research team and grouped according to the emergent themes, ideas and concepts. Two members of the research team analysed the data using TA, a qualitative data analysis technique used ‘for identifying, analysing and interpreting patterned meanings or themes in qualitative data’.^21^ ^,p.79^ Through systematic and transparent coding of the key themes and methodical and honest reporting of the findings, the researchers sought to try to minimize bias. The findings were checked against some of the wider contextual issues and preliminary themes coming out of the focus groups in some of the more in‐depth interviews with practitioners. These components were then re‐evaluated, regrouped as necessary and gradually refined and linked to other conceptual categories. To ensure rigour, the key themes and components coming out of the analysis were initially analysed by the two researchers separately and then brought together to compare and refine. The researchers attempted to maintain a high level of thoughtful and deliberate planning throughout the AI process, and a diligent and ongoing application of researcher reflexivity, as well as honest communication between the researchers, and the participants in the AI process through the Step 3 workshops. The preliminary findings were also discussed with the steering group (*N* = 17) made up of both professionals and members of the public with experience in undertaking co‐production, and/or with some knowledge of the context of the two ActEarly field sites. The steering group provided regular oversight and feedback on the AI process and helped to reduce and draw out any researcher influence on the results.

### 2.5 Step 5: *Deliver*

A strategy and practical guidance were developed and disseminated to the ActEarly consortium and wider stakeholders.

## 3 RESULTS

The findings from the AI process identified nine main themes that characterize effective co‐production (referred to hereafter as principles of co‐production) and three core values. The following sections set out the key themes from the analysis of the data from the focus groups and interviews, the dream and design workshops and other aspects of the AI research. Direct quotations are presented as being from a focus group (FG) or interview (INT), and then whether the stakeholder is in Tower Hamlets (TH) or in Bradford (B).

### 3.1 Principles of co‐production

**Figure 3:**
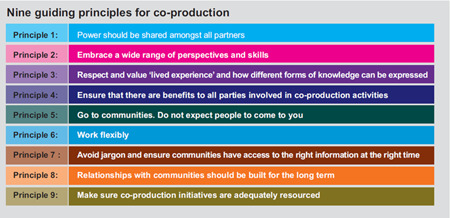
ActEarly co‐production guiding principles

The AI process generated nine guiding principles on how to deliver successful co‐production which are depicted in Figure 3, and then further described in more detail below.

#### 3.1.1 Principle 1: Power should be shared amongst all partners

Successful co‐production requires a recognition of the imbalanced starting positions faced by many of the stakeholders in co‐production. There needs to be an intentional effort from those who hold power to show that they are willing to share it and a focus on building towards that sense of equality.

It needs a lot of groundwork to prepare organisations to share their power—people who currently hold the power need to be committed to co‐production process. (FG3, TH)

With young people we are always aware that … we have more power because we're older but we have to deliberately make a choice to … really listen to what they're saying because it's so easy to, because we're all older, lived longer, to dismiss some of the things that they say. (FG10, B)

#### 3.1.2 Principle 2: Embrace a wide range of different perspectives and skills to ensure these are represented in the project

Nearly all participants in the AI process discussed the importance of ensuring that communities feel properly heard and listened to, which is linked to the theme of building relationships and developing trust (Principle 8).

Get up in to the communities and real groups to better understand the truth, and properly listen to people and what they're saying about what the issues are. (FG8, B)

Everything that's gone well so far has been because we've listened to the mothers, we've understood the mothers, and we've taken forth their ideas, therefore they've felt as if that they have been involved, they've been listened to, they are instrumental in making change. (INT16, B)

Many participants mentioned the importance of partnership working, whether that entails larger, more established and better‐resourced organizations working with smaller ones, or just different stakeholders and partners working together to affect change. The success of partnership working seems to come from the credibility and trust that established relationships can bring, as well as the sharing of resources, and also the focus and balance that such partnerships can bring.

Better collaboration between participants and stakeholders—without it, the work loses focus and balance. From a participant's point of view, they don't care about difficulties communicating between. (FG3, TH)

Working with local community organizations as gatekeepers can help to ensure access to a diverse range of community views, but some participants mentioned the need to be more ambitious and move beyond working solely with a small group of gatekeepers to involving a wider audience. A particular suggestion involved working with designated community champions or community partners to deliver successful co‐production:

It really helps to have a lot of champions in the community—they have unbounded energy, resilience and capacity. Recognising those people and bringing them together has worked well. …In some communities, projects need to be endorsed by the right community members to really work. (INT15, TH)

The key is to have localised grassroots partners and to make sure the focus and needs identified are based in the community and tap into local expertise, not a parachuting‐in viewpoint. Having a grassroots bottom‐up perspective is key in anything of this sort. (INT14, B)

#### 3.1.3 Principle 3: Respect and value of lived experience and how different forms of knowledge can be expressed and transmitted

Participants drew attention to the importance of respecting and valuing the ‘lived experience’ held by different people, and how different forms of knowledge can be expressed and shared.

People can connect if they see different people involved and committed to the work. Different sectors are represented, and you feel that your voice is being heard and your experiences are valued. (FG3, B)

The ways in which knowledge is valued reflects how it is produced, meaning that in many cases, members of the public have been limited to the role of participants in research studies, where data are collected about them, without their influence in the design, data collection or eventual output. However, approaches such as citizen science and co‐production aim to change this. An active and well‐engaged Patient and Public Involvement group in Bradford explained how they support researchers by helping them consider practical and ethical issues from a community perspective. Whilst such inclusion should be celebrated, very few examples came forth to demonstrate the more advanced forms of inclusion whereby community members produce knowledge and conduct research with professionals as a partnership. Several contributors made a case for better inclusion of different forms of knowledge and lived experience to feed into research systems, and how damaging it can be if this is not the case.

I think the worst thing from the cohort … that we work with is to hear them and not listen to them and not follow through with something they're doing, because that's how you cause that disengagement again … because they're going to think that no‐one takes their ideas or experiences into account. (INT12, B)

The benefits of drawing on a plurality of sources and types of knowledge include improved validity and better engagement from target communities. This is possible because local knowledge is the mundane, yet expert understanding of, and practical reasoning about, local conditions derived from lived experience. When the lived experience is pressed into service, using the right approaches, it has the potential to augment research and implementation efforts.

#### 3.1.4 Principle 4: Ensure that there are benefits to all parties involved in co‐production activities

Ensuring that communities receive equal benefits was mentioned often in discussions with stakeholders throughout the AI process. Too often, those leading a co‐production project make things work for their own purposes (e.g. to complete a Public Patient Involvement exercise or to complete the development of a funding bid), and those being engaged do not receive anything in return. This can erode trust, meaning that participating groups are used as a resource in the materialistic sense and not as an asset to help agencies achieve improvements. One way to ensure participating groups benefit in their contribution to a project is to provide visible feedback, with clear pathways to feed into policy. This can be achieved through, a clear and consistent feedback loop that articulates what people expressed during the project, the project team responses and actions, in addition to a commitment for partners to take forward the outputs of projects.

If there is no feedback, people will question the point and not want to be engaged in the future. There has to be a feedback loop explaining to people, for example, why change didn't happen. (INT6, TH)

Representation of different communities is needed since this will give confidence to communities that they are being taken seriously … If they don't see things change then they can't be bothered to contribute. Change breeds confidence. (FG2, B)

#### 3.1.5 Principle 5: Go to communities. Do not expect communities to come to you

Participants also mentioned the importance of going to where people and communities are, rather than expecting them to come to researchers. Equally, talking to people in their own environments informally was seen as a good way to connect. Some participants framed successful co‐production as being about keeping motivated and connected to the direction those participating in the project want to go in and being mindful of giving support when needed and stepping back where space is needed for growth. Such requirements necessarily require a flexible disposition and successful co‐production is an ongoing dialogue with communities and not a one‐off event.

Practice through research … it's about coming along to things already set up to not duplicate or re‐invent the wheel. It's about coming out to groups—it's better to meet in their environments—so services meeting communities halfway—currently it's a very top‐down approach with little co‐production with communities … We need to change the way we work, be more practical and do things differently. (FG11, B)

#### 3.1.6 Principle 6: Work flexibly

Working flexibly, including adapting activities to be able to work both face‐to‐face and online as appropriate, and talking to people in their own environments in informal ways was seen as crucial to successful co‐production. For this to work well, it is necessary to pay attention to the varying timescales of different sectors and organizations, as well as the importance of involving communities from the beginning rather than including some co‐productive element to a project as an add‐on further down the line. Alignment of competing timescales can be challenging due to different priorities and foci and can be further intensified by political cycles, which can force timescales in different directions. Working flexibly also includes learning through trial and error, and being open to adapting and changing approaches and methods used in a project:

Sometimes people can make things too complicated as they're sticking to a formular rather than adapting to context … with co‐production you have to change as you go along, and you have to be open to change your methodology. (INT6, TH)

#### 3.1.7 Principle 7: Avoid jargon and ensure communities have access to the right information at the right time

All those involved in a project need to have access to information in the appropriate cultural context for them, such as the appropriate language, and to be clear about what is being aimed for in a project. Using the appropriate terminology and language is crucial to the feedback process, to ensure participants understand how a project has evolved and if or how changes have been adopted. Reflecting on the language used in co‐production—particularly where multiple professions and stakeholder groups are involved—cannot be underestimated, since each profession or group will have their own terminologies and ways of framing or talking about particular issues, based on their own experiences and other contextual factors.

We need to use appropriate language for different stakeholders and service users to explain what we're doing. (INT15, TH)

Professionals use language that can exclude people—like acronyms. People feel out of place if they ask things to be explained to them. The language of co‐production involves an understanding that participation for residents or communities is harder as people don't feel confident. It's important to avoid using terminology or acronyms that might make this worse. (FG2, B)

Avoiding jargon and using more collaborative terms, such as aims, values and ethos, can help to resolve potential conflict due to the lack of shared vision. However, there needs to be as much clarity as possible from the outset of a project around what is being aimed for, to avoid a ‘drift’ in direction, and to keep things moving towards the desired goals of all involved. Participants warned of overpromising on outcomes and a lack of clarity about what is on the table from the outset.

#### 3.1.8 Principle 8: Relationships with communities should be built for the long term and not for the short term

Participants noted the importance of the level of trust communities have in both the process and the people delivering activities. This was seen as a deal‐breaker as people have seen many ideas come and go and noticed who makes a difference and which services are not actively making a difference. The act of doing things together builds on this level of trust and creates an opportunity for services or research projects to return in the future to capitalize on this trust:

Community events and group activities work well because they build relationships, they continue to build relationships—so there's an element of relationship building within the activity that you're doing. (FG10, B)

Building individual relationships even when convening groups, or largely doing group work is really important. It's about knowing where people are coming from, how best to support them. The human approach and connection with people is very important. (FG3, TH)

Participants mentioned that communities feel disengaged and frustrated when research teams ‘parachute’ in to work with them on short‐term projects, and then leave without actioning community priorities, but funders often work on short‐term funding cycles, and this mismatch in timeframes produces its own challenges. Participants highlighted how co‐production works best when it is focused on tangible action and when people can see how their contributions have been followed up.

Co‐production works best around tangible action—people enjoy contributing to things they are seeing develop in front of their eyes. Some things have different timescales. (FG3, TH)

Our strength has always come from the fact that if they've said something as random as it may sound, we follow through with it even if it costs money, but that's how we've gained the trust of them because we follow through. (INT16, B)

#### 3.1.9 Principle 9: Make sure co‐production initiatives are adequately resourced

The topic of adequate time and resources to do co‐production successfully was frequently discussed. Building strong links with the voluntary and community sector is important, but this takes time and funding for all parties involved. Participants raised issues with funding specifically for co‐production, suggesting that it is challenging to appropriately allocate time and resources between co‐production and other mechanisms for engagement and priorities. They also mentioned the issue of capacity, and that it takes time for everyone to be in the same place, which is exacerbated by the way research funding works—with short timelines, insufficient budgets, and there is always an added extra that gets cut back.

To make a meaningful difference … [co‐production] has to be built into commissioning and grant making, so that people are resourced to be able to work in that way. (INT12, B)

You can often fall into trap of chasing funding based on perceived need, rather than working with community to identify what's really needed. (INT14, B)

A competitive environment for the voluntary sector in terms of seeking funding can lead to a resistance to sharing data and collaborating which poses a challenge to notions of partnership working and reciprocity raised above. There is also a very real danger of not recognizing the labour that goes into doing co‐production which means participants are not properly rewarded for their time and intensive work.

### 3.2 Dream workshops

**Figure 4:**
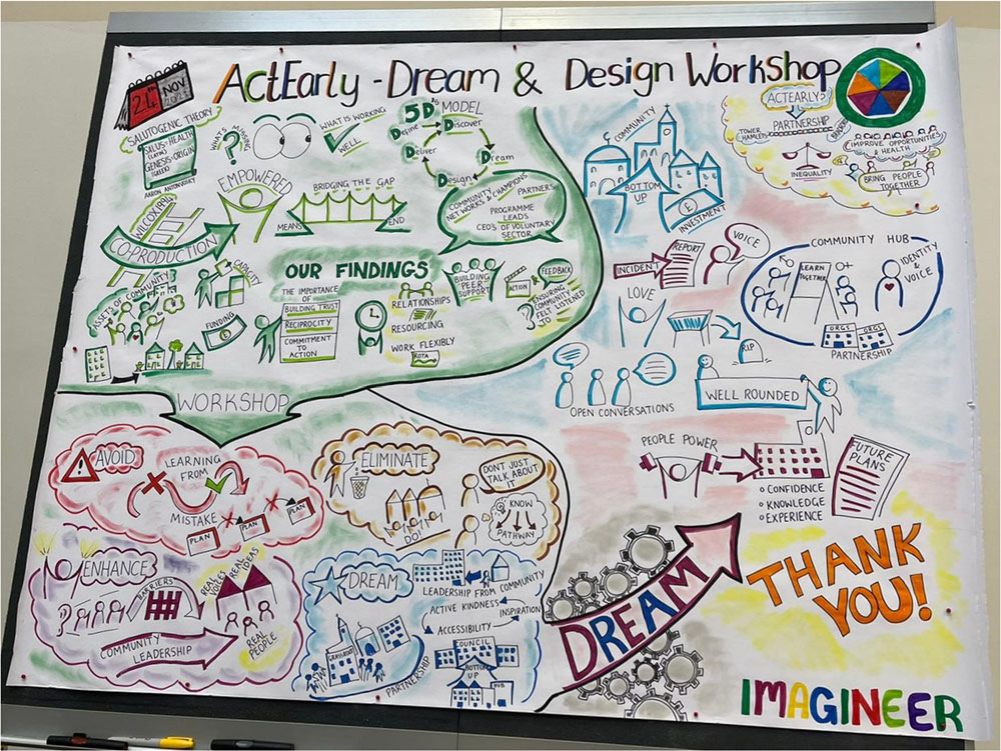
Dream workshop in Bradford graphic summary

**Figure 5:**
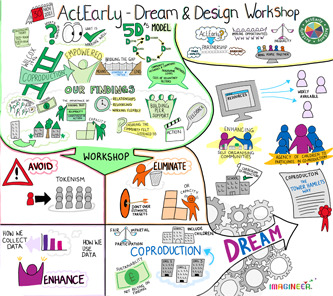
Dream workshop in Tower Hamlets graphic summary

The discussions during each workshop were captured by a graphic illustrator, and the outputs are shown in Figures 4 and 5. below. The outputs from these events were used to assist the research team in formulating the actions to be taken as a result of the AI process.

In the Bradford workshop, participants recognized the politics and changing power dynamics at play between different actors, stakeholders and organizations. They highlighted the importance of building long‐term commitments and building on existing knowledge and expertise that already exists in communities, whilst asking for political decision‐making to be representative of the communities being served. They discussed the possibility of developing a community‐led co‐production hub as a shared learning environment, a place for all ages and communities to come together, and a space for agencies to be ‘guests’ in a community space. Participants also discussed the importance of investing in diverse community champions to coordinate a bottom‐up approach to responsibly using resources effectively and to procure for social value, whilst also recognizing the limitations of what is possible.

In the Tower Hamlets workshop, discussions focussed on ensuring and developing co‐production capacity within organizations that is sustainable and for the long term; identifying opportunities and repurposing existing structures and mechanisms to promote co‐production and training professionals to see co‐production as an integral part of day‐to‐day activities. The discussion also explored the potential to include children as co‐producers, and the development of a recognized approach to co‐production in Tower Hamlets, or an awards scheme and celebration of successful co‐production activities.

### 3.3 Core values underpinning co‐production

Through reflection and dialogue with all stakeholders in various forms throughout the AI process, three core values emerged that underpin the principles stated above that make co‐production distinct from other forms of inclusive approaches. These values demonstrate what co‐production is comprised of.

#### 3.3.1 Equality

All those participating in co‐production must feel they are equal contributors to the process of design and delivery, and this requires inclusion from the very start of a project, all the way through to evaluation. Equality does not mean treating everyone the same but necessarily focuses on treating participants differently by respecting and accommodating their difference.

#### 3.3.2 Agency

Respecting the goals and values of community members may well be (and probably will be) different to the organizations that have commissioned a project. Finding ways to respect this and negotiating and accommodating this difference is a crucial underlying value of successful co‐production.

#### 3.3.3 Reciprocity

This refers to the mutually beneficial exchange of knowledge and resources in a context of partnership, where all parties have something to gain. This will be different for each party, but this must be expected and respected.

### 3.4 Co‐producing the co‐production strategy

Together the nine principles and three core values form the underpinnings of the co‐production strategy to guide the ActEarly consortium. Together with the steering group overseeing the process, a set of specific actions or practical suggestions was developed to take forward the principles and values of the co‐production strategy (see Supporting Information: File 1).

## 4 DISCUSSION

This paper reports on the process of co‐producing a co‐production strategy for a consortium of researchers, practitioners, stakeholders and communities who aim to implement system‐based approaches to improving children's health in two locations in England. The co‐production strategy also includes a list of practical suggestions for researchers and practitioners wishing to implement the values and principles of the co‐production strategy, recognizing that some principles will be easier to enact than others. The principles developed through the AI process are consistent with previous work to capture the foundational principles of co‐production. For example, Harrison et al's.^22^ narrative reviews identify, quantify and summarize the conceptual foundational principles of patient stakeholder engagement in research and best practice activities and found that the most commonly reported foundational principles were ‘respect’ and ‘equitable power between all team members’. For co‐production to flourish, attention needs to be turned to appropriate, long‐term resourcing of community assets (e.g., community centres, schools and faith settings) that can act as ‘gate‐keepers’ to communities, and also for the researchers and services who need to work with communities.^23^ Smith et al.^4^ draw attention to the fact that researchers operationalize co‐production in various ways, often without the necessary financial and organizational support required and the right conditions for success. Projects ‘parachuting’ in to work on specific topics and then leaving after that project ended, or those not appearing to fulfil community needs, were seen as very damaging to trust.^24^ It would seem prudent to develop appropriate community infrastructure at a ‘place’ level, providing hubs that can connect communities with researchers and stakeholders and provide opportunities for longer‐term dialogue. Central to this longer‐term dialogue is allowing communities to be the driving force in the identification of their own priorities, and that funders should tailor their commissioning cycles to suit. Experience of priority setting in Bradford,^25^ under the umbrella of the ActEarly programme has found that communities identify a range of issues important to children's health and happiness and that with commitment and joint working, these can be translated into shared research and service agendas. This is also supported by previous work to capture the core principles in co‐design and research, which have found consistent results.^22^, ^26^ Following the development of our co‐production strategy, the ActEarly research team is developing specific actions to progress opportunities for dialogue with communities. These include a commitment to organize feedback sessions in Bradford and in Tower Hamlets, through regular open space meetings in each location, as an opportunity to check in, to share information about what projects are taking place, and also to share knowledge about what has worked, and areas where things have gone less well. Such meetings could also serve as an opportunity to feed into an evaluation of co‐production—to review whether the process worked in the way it was intended, and if not, why not. These sessions would be dedicated spaces for reflecting on what was produced, what worked and what did not—a factor that is particularly important to ensure participants do not feel that it is their fault if a process or activity has not worked out. This is consistent with Witteman et al.'s^26^ offering pragmatic, actionable lessons for developing effective research partnerships between different stakeholders such as patients, caregivers, clinicians and researchers.

Another outcome of the AI process is the need to better understand when co‐production has worked well, and what are the criteria for its success. Furthermore, appropriate attention and consideration need to be given to monitoring, data capture and evaluation, and to reflect on how routine data is gathered, how well it is gathered and how any change affected is different as a result of having used co‐production.^3^, ^13^ Participants warned against too many metrics since communities work at their own pace, and there is a need to reduce pressure on monitoring reports with too many targets and outcomes set by external partners. Concomitant with this, there is a need to focus on long‐term and sustainable outcomes that build on trusted relationships and continue long after a project or piece of research has finished.

### 4.1 Strengths and limitations

A strength of the approach used to co‐produce a co‐production strategy was the use of an asset‐based AI approach to include a wide range of stakeholders and communities in its development.^2^ This process, in and of itself, strengthened relationships between stakeholders and has built on existing community knowledge and assets, enabling the research team to base the strategy on enhancing existing approaches, rather than re‐inventing the wheel. AI practices change the character of interpersonal interactions, including changing perspectives, focusing upon and learning from past successes, and forming relationships and a common vision.^19^, ^27^ Furthermore, the use of the AI process meant that the co‐production strategy developed is grounded in research conducted at a local level and represents an articulation of how the stakeholders and local groups in the two ActEarly sites understand themselves and their work.

The key audience for this work is a multidisciplinary group of researchers, theme leads, policy makers and other interested parties that make up the ActEarly consortium. Perhaps the most challenging aspect of the strategy was to try to reconcile the differing perspectives of the consortium stakeholders with what the community groups in each location articulated to the research team. Further to this, in some instances, there were some differences of opinion that could not be reconciled. This, however, underlines the importance of bringing together diverse groups and stakeholders so that they might work together to develop projects and services and approaches that are acceptable, and feasible. The strategy was developed in two places with similar, high levels of deprivation and ill health. The parallels of opinions across the two sites were striking, however, it may be that other ‘places’ have different opinions and priorities. An outcome of this strategy development work is to encourage all those undertaking co‐productive activities to engage with their communities at the start to set the principles and underlying values of the process, perhaps using the strategy developed here as a starting point. For example, Fleming and Rhodes^28^ discuss the challenges of embedding lived experience in research, and the NIHR^17^ guidance on co‐production highlights the assumption implied in co‐production that those with lived experience are often best placed to design and deliver research. The challenge lies in actually acknowledging this lived experience and embedding it in the research process.

## 5 CONCLUSION

Effective co‐production necessitates a level of commitment, proper resources, openness to work flexibly and to listen and reflect on shared dialogues and priorities. It requires appropriate resourcing and infrastructure, and a long‐term vision. Whilst co‐production presents many opportunities, it is crucial to recognize that there is not a ‘one size fits all’ approach, and there is a critical need to accept the diversity in approaches to co‐production, and to better tailor these approaches to context, different stakeholder groups and the different stages of the research and implementation process. This requires extensive reflection on the use of chosen approaches in practice and a more systematic reporting process on the learning that ensues. This includes where processes did not work out, failures and areas for improvement. Many might find putting the principles and values outlined here into practice daunting if they do not consider themselves to be ‘skilled’ at co‐production. The aim of this paper is to invite others to read the ActEarly co‐production strategy and find both explanations and practical guidance on how to put co‐production into practice. By providing a set of principles, along with practical recommendations, others can find ways to reflect on their current practice and explore new ways of working and find ways of improving co‐production activities they are involved with. The paper constitutes an invitation to others to build on the methods and findings presented here, with the overall aim of allowing for the better operationalization of co‐production principles and to guard against the tokenistic use of, and potential hollowing out of, the term ‘co‐production’.

## CONFLICT OF INTEREST

The authors declare no conflict of interest.

## ETHICS STATEMENT

The project was reviewed and received ethical approval from the University College London Research Ethics Committee ID 19659/001.

## Supporting information

Supporting information.

## Data Availability

The data collected as part of this research that support the main findings of this study are not available for reuse. Data are available on request due to privacy/ethical restrictions.
