# Supplementary material for: Nothing about us without us: A co‐production strategy for communities, researchers and stakeholders to identify ways of improving health and reducing inequalities
Source: Health Expect. 2023 Jan 22;26(2):836–46. doi: 10.1111/hex.13709 (PMC10010091; doi:10.1111/hex.13709)
Supplement: Supplementary file 1 — Supporting information. [file HEX-26--s001.docx]

**HEX paper supplemental file 1**

| **No** | **Principle title** | **Suggested action** |
| --- | --- | --- |
| 1. | Sharing of power | Aim to keep membership of a working group stable, with a balance of backgrounds. For example, if there are likely to be many professionals in the group, then increase members of the community to improve the balance. Having multiple people provides confidence as people will not feel like a ‘lone voice’ and can discuss their ideas with each other. |
|  |  | Professional colleagues should make time to speak to community members outside of meetings to clarify matters. For example, some points raised in meetings may be very familiar to professionals but may be new and unfamiliar to community members. |
|  |  | Rotate the roles during meetings and change who chairs the meetings. When appropriate to do so, the chairing should be made available to community members with the right level of support. |
|  |  | Change how a room is set up to make sure people feel comfortable – it might be helpful to ensure that no clusters are formed of any groups especially avoid professionals sitting in one area and community members in another area. |
|  |  | Use more group work and other techniques such as sharing stories and have fewer formal presentations. This will make it easier for people who do not like speaking in front of large audiences to express their opinions more easily |
| 2. | Including all perspectives and skills | Make use of creative approaches that suit communities such as open-space approaches, art-based workshops, and other similar methods that make it possible for people to contribute their perspectives and skills. |
| 3. | Respecting and valuing knowledge and lived experience | To ensue different types of knowledge are incorporated requires a shift in how we value community knowledge and make use of it. |
|  |  | Experiential and presentational knowledge are vital ingredients of co-production. Community members need to be informed about the value of this. |
|  |  | Appropriate systems will need to be put in place to encourage people to contribute their knowledge – for example creative approaches such as art-based methods. |
|  |  | It is important to ask community members outside of meetings if the system for their inclusion allows them to contribute their knowledge and what could be done differently, if anything, to improve this further. |
| 4. | Reciprocal benefits for all | Be clear from the outset about the anticipated timescales for impact and communicate these to community members. However, do not over-promise. |
|  |  | Ensure communities are reimbursed appropriately for their input using your local payment and reimbursement policy. |
|  |  | Work with communities to explore what additional benefits there might be to engaging in co-production activities. For projects with more intensive, longer-term input there could be opportunities for learning new skills or for volunteering. For less frequent activities it could be ‘getting a foot in the door’ for future opportunities. There is no one-size-fits all approach and different communities will expect and want different things. |
|  |  | If community organisations become energised about the topic and wish to seek grant funding from external sources to improve a matter that you have worked on together then provide assistance with the process (if needed) to achieve this. |
| 5. | Going to communities and not expecting them to come to you | Researchers should use a variety of methods to engage communities and the methods used for co-production should fit the communities rather trying to find a method that works best for the organisers. |
|  |  | Whenever possible researchers should visit the communities they are doing research with to improve connectivity and understanding by being present and also to possibly increase the chances of more people coming forward to support co-production approaches. |
| 6. | Working flexibly | Researchers should use a variety of methods to engage communities in co-production activities and it is important to stress that co-production is not a single tool but should instead be viewed as a toolbox. |
|  |  | Researchers should be prepared to flex their co-production plans and recognise that plans may change and evolve. |
|  |  | Communities are not beholden to project plans or timelines and work needs to be done at a pace suitable to the community’s capacity. |
| 7. | Appropriate terminology and information | Avoid using jargon or acronyms. |
|  |  | Making use of existing communication systems that are familiar to the community we intend to work with can improve responsiveness. For example, WhatsApp groups or existing communities of interest groups that are united through social media platforms. |
|  |  | In order to ensure that all groups feel included, it may be necessary to produce materials and run co-production activities in relevant community languages. |
|  |  | Researchers should explore use of translators, or local community organisations who have the skills and can possibly create a bridge to the communities. |
|  |  | Ensure that information about research projects is produced in ‘plain English’. Ask community members to help review documentation. |
|  |  | Using the appropriate terminology and language is equally crucial at the feedback stage. This is to ensure all those involved can see and appreciate the output of their co-productive efforts. |
| 8. | Building and maintaining long term relationships | Effective relationship building with communities needs to be prioritised within research and commissioning organisations. |
|  |  | Effective engagement infrastructure needs to be developed; and maintaining this central engagement infrastructure should be a core part of everybody’s role within the research programme. |
|  |  | Local organisations, for example voluntary sector charities or faith settings, can be effective gatekeepers to accessing community voices and for that reason strong relationships should be developed with these organisations. |
|  |  | Regular communication and shared priority setting will be crucial to the long-term success of this venture. |
|  |  | Research teams should work with local organisations to identify and progress partnership approaches. |
| 9. | Adequate resources | Researchers applying for grant funding should consider including local organisations as co-applicants and include adequate funds for them to contribute in a co-productive way. |
|  |  | Research teams should co-ordinate at a departmental and institutional level to co-ordinate community engagement approaches and ensure a consistent approach is adopted, and communities are not over-burdened. |
|  |  | Researchers should aim to establish links with local organisations relevant to their programmes of work and communicate regularly about priorities, plans and progress. |
|  |  | Regular community engagement activities should be planned either using face to face or online methods and all members of the research team should take an active role in facilitating these. |
